# Supplementary material for: Healthcare seeking behavior and delays in case of Drug-Resistant Tuberculosis patients in Bangladesh: Findings from a cross-sectional survey
Source: PLOS Glob Public Health. 2024 Jan 24;4(1):e0001903. doi: 10.1371/journal.pgph.0001903 (PMC10807832; doi:10.1371/journal.pgph.0001903)
Supplement: Attachment — Submitted filename: Response to Reviewers.docx [file pgph.0001903.s002.docx]

| S.No | Comment | Response |
| --- | --- | --- |
| Journal-related comments | | |
| 1. | 1. Fig 1: please (a) provide a direct link to the base layer of the map (i.e., the country or region border shape) and ensure this is also included in the figure legend; and (b) provide a link to the terms of use / license information for the base layer image or shapefile. We cannot publish proprietary or copyrighted maps (e.g. Google Maps, Mapquest) and the terms of use for your map base layer must be compatible with our CC-BY 4.0 license.  Note: if you created the map in a software program like R or ArcGIS, please locate and indicate the source of the basemap shapefile onto which data has been plotted. | Thank you for your feedback. For 1(a), Now, we have added a direct link to the base layer of the map in the figure legend. (line number 178, 179), also stated clearly in the methodology section (line number 254-256)For 1(b), We have reproduced Figure 1 using a shapefile from a freely available database (GADM; www.gadm.org). We also reference the source of the shapefile (line numbers 616 and 617). The terms of use for our map base layer are compatible with CC-BY 4.0 license as GADM; www.gadm.org clearly stated that on their website.  We have also attached a consent email response from GADM with our response. |
| 2. | Please include a complete copy of PLOS’ questionnaire on inclusivity in global research in your revised manuscript. Our policy for research in this area aims to improve transparency in the reporting of research performed outside of researchers’ own country or community. The policy applies to researchers who have travelled to a different country to conduct research, research with Indigenous populations or their lands, and research on cultural artefacts. The questionnaire can also be requested at the journal’s discretion for any other submissions, even if these conditions are not met.  Please find more information on the policy and a link to download a blank copy of the questionnaire here: <https://journals.plos.org/globalpublichealth/s/best-practices-in-research-reporting>. Please upload a completed version of your questionnaire as Supporting Information when you resubmit your manuscript. | Thank you for the comment. A copy of the requested document has been attached. |
| 3. | Some material included in your submission may be copyrighted. According to PLOS’s copyright policy, authors who use figures or other material (e.g., graphics, clipart, maps) from another author or copyright holder must demonstrate or obtain permission to publish this material under the Creative Commons Attribution 4.0 International (CC BY 4.0) License used by PLOS journals. Please closely review the details of PLOS’s copyright requirements here: PLOS Licenses and Copyright. If you need to request permissions from a copyright holder, you may use PLOS's Copyright Content Permission form.  Please respond directly to this email or email the journal office and provide any known details concerning your material's license terms and permissions required for reuse, even if you have not yet obtained copyright permissions or are unsure of your material's copyright compatibility.  Potential Copyright Issues:  Figure 1: please (a) provide a direct link to the base layer of the map (i.e., the country or region border shape) and ensure this is also included in the figure legend; and (b) provide a link to the terms of use / license information for the base layer image or shapefile. We cannot publish proprietary or copyrighted maps (e.g. Google Maps, Mapquest) and the terms of use for your map base layer must be compatible with our CC-BY 4.0 license.  Note: if you created the map in a software program like R or ArcGIS, please locate and indicate the source of the basemap shapefile onto which data has been plotted.  If your map was obtained from a copyrighted source please amend the figure so that the base map used is from an openly available source. Alternatively, please provide explicit written permission from the copyright holder granting you the right to publish the material under our CC-BY 4.0 license.  Please note that the following CC BY licenses are compatible with PLOS license: CC BY 4.0, CC BY 2.0 and CC BY 3.0, meanwhile such licenses as CC BY-ND 3.0 and others are not compatible due to additional restrictions.  If you are unsure whether you can use a map or not, please do reach out and we will be able to help you. The following websites are good examples of where you can source open access or public domain maps:  * U.S. Geological Survey (USGS) - All maps are in the public domain. ([http://www.usgs.gov](http://www.usgs.gov/))  * PlaniGlobe - All maps are published under a Creative Commons license so please cite “PlaniGlobe, [http://www.planiglobe.com](http://www.planiglobe.com/), CC BY 2.0” in the image credit after the caption. (<http://www.planiglobe.com/?lang=enl>)  * Natural Earth - All maps are public domain. (<http://www.naturalearthdata.com/about/terms-of-use/>) | Thank you for your remark. For Figure 1, we created the map in a software program ArcGIS v10.8 and the shape files were downloaded from free online sourse <https://www.diva-gis.org/gdata>.  The information regarding the software and source of the shape file are now added in the methodology section. |
| Comments to the Author – Reviewer 1 | | |
| 1. | Introduction: The introduction needs refinement in both sense, scientifically and grammatically. Drug-Resistant section definitions should be updated like MDR-TB, XDR-TB latest definitions should be incorporated. | Thank you for the suggestion. The introduction is now refined both scientifically and grammatically. The definition of MDR-TB and XDR-TB are updated according to the latest information. |
| 2. | Methodology: Operational section in methodology should be revised (like definitions of new, re-treatment and relapse cases). | Thank you for the suggestion. The operational definitions of new, re-treatment, and relapse cases are now revised according to the latest update. |
| 3. | Result section should be reviewed as mentioned in text majority of the participants are male while in table-3 it’s mentioned as female.  The citations of tables/figures, should be uniform.  Results discussed should be same both in text, tables and figures. | Thank you for the suggestion. The result section is reviewed and necessary corrections are made in table-3.  The citation of table/figures are reviewed and necessary corrections are made to keep it uniform.  Necessary corrections are made to keep the result section uniform both in text, table, and figures. |
| 4. | Include literature review from recent studies about the topic if available. | Thank you for the suggestion. Literature reviews from some recent studies are now added in the discussion section. |
| Comments to the Author – Reviewer 2 | | |
| 1. | The authors talk about the End-TB 2030 strategy, but it is not clear where this strategy comes from. Please briefly give details about it.  - On page 5, from lines 98-99 to me, healthcare-seeking behaviour is not an influential factor. As indicated, it is a behaviour/tendency about accessing and utilising healthcare in response to given ailments, and it is typically shaped by the contexts in which human beings live (i.e., socioeconomic circumstances, beliefs about health, stigma, accessibility). The statement, therefore, needs to be conceptually correct.  - I noticed several terms have been interchangeably used to refer to the same thing; for instance, what is the difference between drug sellers/pharmacists OR traditional healers/traditional providers/informal providers/ayurvedic practitioners OR village doctors/doctors? Please be specific and consistent about using these terms, as a lack of clarity in them can confuse the reader in understanding the analysis. | Thank you for the suggestion. A brief detail about the End-TB 2030 strategy is added to the introduction.  The statement on page 5, lines 98-99 is now rewritten, and other potential determinants that are typically shaped by the contexts in which human beings live (i.e., socioeconomic circumstances, beliefs about health, stigma, accessibility) are now included.  The necessary changes are made to be specific and consistent about using terms. For example, all pharmacists are replaced by “drug sellers”. Also now other terms also use specifically and consistently to ensure the readers have a clear understanding of the analysis. |
| 2. | Methods:  - Structured questionnaires are tools that help collect data about various variables. They do not help to conduct “face-to-face interviews”. The term face-to-face interviews transmit a message about a qualitative design, which was not used in the study. Maybe you write as “a pretested structured questionnaire was used to gather quantitative data OR data about XYZ”. | Thank you for the suggestion. The method is rewritten excluding the term “face-to-face interviews”. |
| 3. | Results:  - Page 12, line 242: “Most of the patients 42 (45.65%) had never attended school”. 45% is “less than half” of the total sample, and it should be written accordingly. Please make changes to other similar descriptions, with appropriate classifications: few (2-5), a few (6-15), some (16-30), many (31-39), less than half, more than half, and most/majority of etc – just to give an example to come up with criteria to consistently describe the numbers. Logical descriptions are very important when the quantitative data is explained. Please look at all the numbers logically/objectively.  - How did you ask about “Haemoptysis” and “anorexia” from patients? I suspect that this is the authors’ conceptualization of the symptoms reported by the patients. Perhaps, simplify them as asked via the questionnaire or told by the patients (cough with blood, for instance). | Thank you for the suggestion. The description of the result section is revised according to the suggestion.  The term “Haemoptysis” is now replaced by “cough with blood” and the term “anorexia” is replaced by “loss of appetite” |
| 4. | Discussion:  This section typically allows authors to reflect on findings and tell the readers what is meant by what they have found (in the results section). However, most of the content presented in the section is a simple restatement of the findings discussed earlier. It is always useful to restate findings, but limiting it to the initial first paragraph is enough. This section must include some further analysis of why certain segments of the sample were more likely to experience delays than others in terms of seeking care, diagnosis, and treatment. I am aware that the authors talked about factors like informal providers and over-the-counter medication. But there is a wealth of literature in low- and middle-income countries such as India and Pakistan speaking about informal providers’ links with pharmaceutical companies and how to achieve targets, informal providers overprescribe antibiotics. This literature (from Bangladesh and other countries) needs to be included in the analysis.  Additionally, there is not much focus on the study findings’ implications for policy and practice. The authors need to describe how they see the research can help identify gaps in Bangladesh’s health system (regulation, policy, and practice), and what things could be improved within the health system to address the issue of delayed diagnosis and treatment of TB. Also, now that we know that in Bangladesh DR-TB is prevalent, and also some factors are contributing to it, can we speak something about the pharmaceutical industry and how could it play a part to address the issue (i.e., promoting ethical prescribing, refraining to work with informal providers, and focusing more on R&D)? | Thank you for the suggestions. Some further analysis of why certain segments of the sample were more likely to experience longer delays is added in the discussion section. The link between informal providers and pharmaceutical companies and how it enhances the overprescription of antibiotics is added and the literature review from neighboring countries is now included.  A detailed description of how the research findings can help to identify gaps in the Health System of Bangladesh in the aspect of health regulation, policy practices, and what things could be improved within the health system to address the issue of delayed diagnosis and treatment of TB is now added. Recommendations about pharmaceutical companies promoting ethical prescribing, refraining from working with informal providers, and focusing more on R&D are also added in the discussion section. |
